# Submerged fermentation of *Streptomyces uncialis* providing a biotechnology platform for uncialamycin biosynthesis, engineering, and production

**DOI:** 10.1093/jimb/kuab025

**Published:** 2021-03-19

**Authors:** Hindra, Dong Yang, Jun Luo, Tingting Huang, Xiaohui Yan, Ajeeth Adhikari, Christiana N Teijaro, Huiming Ge, Ben Shen

**Affiliations:** Department of Chemistry, The Scripps Research Institute, Jupiter, FL 33458, USA; Department of Chemistry, The Scripps Research Institute, Jupiter, FL 33458, USA; Natural Products Discovery Center at Scripps Research, The Scripps Research Institute, Jupiter, FL 33458, USA; Department of Chemistry, The Scripps Research Institute, Jupiter, FL 33458, USA; Department of Chemistry, The Scripps Research Institute, Jupiter, FL 33458, USA; Department of Chemistry, The Scripps Research Institute, Jupiter, FL 33458, USA; Department of Chemistry, The Scripps Research Institute, Jupiter, FL 33458, USA; Department of Chemistry, The Scripps Research Institute, Jupiter, FL 33458, USA; Department of Chemistry, The Scripps Research Institute, Jupiter, FL 33458, USA; Department of Chemistry, The Scripps Research Institute, Jupiter, FL 33458, USA; Natural Products Discovery Center at Scripps Research, The Scripps Research Institute, Jupiter, FL 33458, USA; Department of Molecular Medicine, The Scripps Research Institute, Jupiter, FL 33458, USA

**Keywords:** Biosynthesis, *Streptomyces uncialis*, Submerged fermentation, Titer improvement, Uncialamycin

## Abstract

Uncialamycin (UCM) belongs to the anthraquinone-fused subfamily of 10-membered enediyne natural products that exhibits an extraordinary cytotoxicity against a wide spectrum of human cancer cell lines. Antibody-drug conjugates, utilizing synthetic analogues of UCM as payloads, are in preclinical development. UCM is exclusively produced by *Streptomyces uncialis* DCA2648 on solid agar medium with low titers (∼0.019 mg/l), limiting its supply by microbial fermentation and hampering its biosynthetic and engineering studies by *in vivo* pathway manipulation. Here, we report cultivation conditions that enable genetic manipulation of UCM biosynthesis *in vivo* and allow UCM production, with improved titers, by submerged fermentation of the engineered *S. uncialis* strains. Specifically, the titer of UCM was improved nearly 58-fold to ∼1.1 mg/l through the combination of deletion of biosynthetic gene clusters encoding unrelated metabolites from the *S. uncialis* wild-type, chemical mutagenesis and manipulation of pathway-specific regulators to generate the engineered *S. uncialis* strains, and finally medium optimization of the latter for UCM production. Genetic manipulation of UCM biosynthesis was demonstrated by inactivating selected genes in the engineered *S. uncialis* strains, one of which afforded a mutant strain accumulating tiancimycin B, a common biosynthetic intermediate known for the anthraquinone-fused subfamily of enediyne natural products. These findings highlight a biotechnology platform for UCM biosynthesis, engineering, and production that should facilitate both its fundamental studies and translational applications.

## Introduction

The enediyne natural products combine unprecedented molecular architectures with extraordinary cytotoxicity against a wide spectrum of human cancer cell lines (Adhikari, Teijaro, Townsend, et al., 2020; Rudolf et al., 2016). All enediynes contain a 9- or 10-membered carbocycle unit with two acetylenic groups conjugated to a double bond or an incipient double bond. While the extreme cytotoxicity has hampered their direct use as systemically administered chemotherapeutic agents, clinical utilizations of enediynes by targeted delivery or as a payload class for antibody-drug conjugates (ADCs) have proven to be highly efficacious for anticancer therapy. Polymer-conjugated neocarzinostatin (SMANCS) and antibody-conjugated calicheamicin (Mylotarg and Besponsa) have both been successfully developed into marketed drugs (Adhikari et al., 2021; Hamann et al., 2002; Lamb 2017; Maeda 2001).

**Fig. 1.**
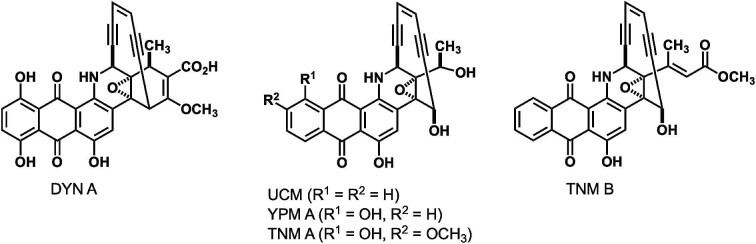
Structures of anthraquinone-fused subfamily of enediynes DYN A, UCM, YPM A, and TNM A, as well as the common intermediate TNM B to UCM and TNM A biosynthetic pathways.

Uncialamycin (UCM), together with dynemicin A (DYN A), tiancimycin A (TNM A), and yangpumicin A (YPM A) form a subfamily of the enediyne natural products with an anthraquinone moiety connected to the 10-membered enediyne core, known as the anthraquinone-fused enediynes (Fig. 1) (Davies et al., 2005; Konishi et al., 1989; Yan et al., 2016, 2017). The similarity in both structures and biosynthetic gene clusters (BGCs) (Fig. 1 and Supplementary Fig. S1) suggests a common biosynthetic pathway for the anthraquinone-fused subfamily of enediyne natural products (Yan et al., 2018). As part of a screening program to identify new antibiotics, UCM was isolated in 2005 from solid agar cultures of *Streptomyces uncialis* DCA2648 with an extremely low titers at ∼0.019 mg/l, produced over a lengthy cultivation period of 2–3 weeks (Davies et al., 2005). Supply of UCM and analogues has relied on total synthesis (Nicolaou et al., 2016). While this has enabled investigations into the structure–activity relationship of UCM and designer analogues as ADC payloads in preclinical studies (Chowdari et al., 2019; Poudel, Naidu, et al., 2020, Poudel, Rao, et al., 2020), access to enediyne natural products for clinical applications has exclusively relied on microbial fermentation (Adhikari, Teijaro, Townsend, et al., 2020).

**Fig. 2.**
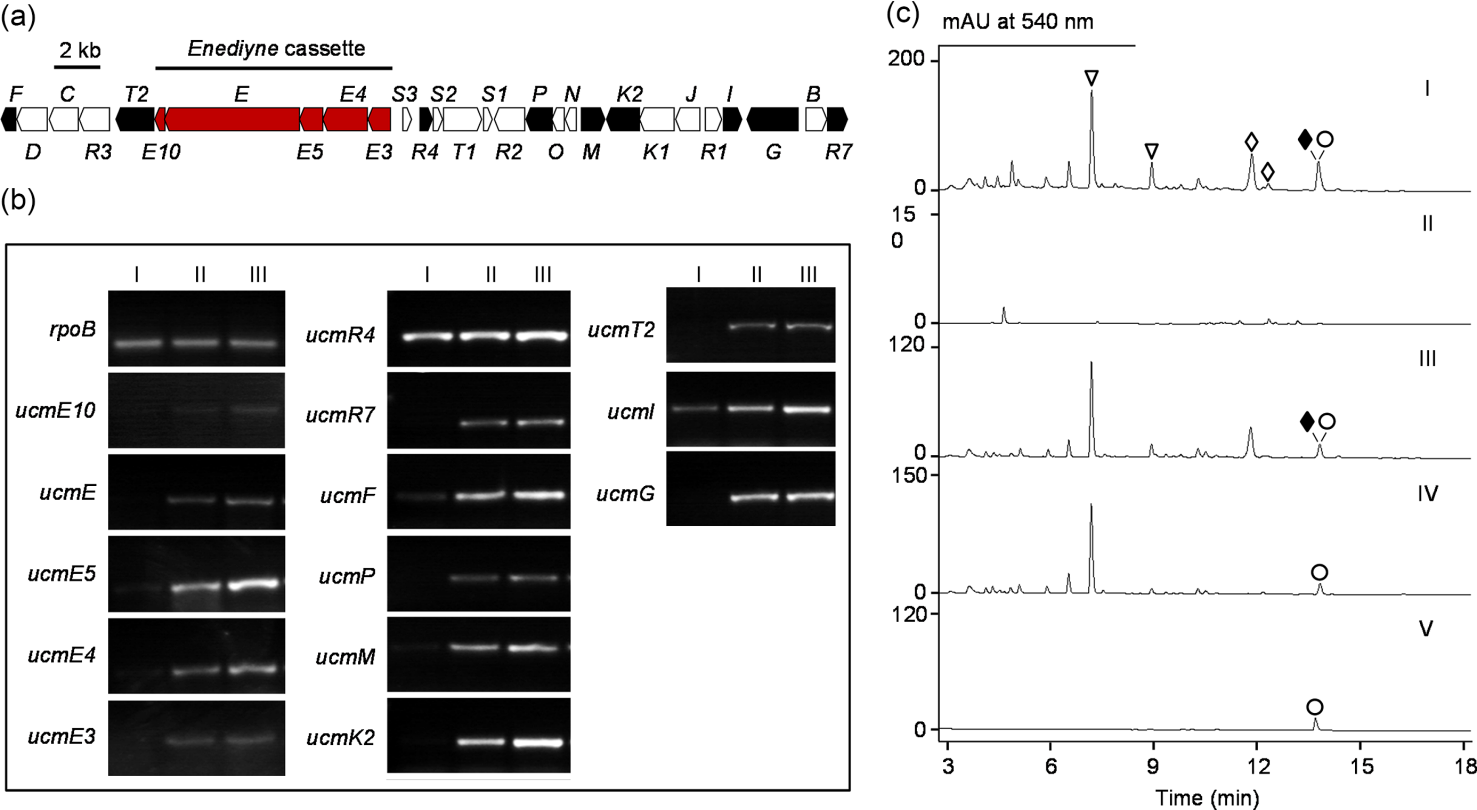
Activation of UCM biosynthesis during submerged growth of *S. uncialis* wild-type and engineered strains. (A) The *ucm* gene cluster from S. *uncialis* DCA2648. Red arrows, enediyne PKS gene cassette; black arrows, representative genes chosen for transcriptional analysis in each putative operon. (B) Transcriptional profiles for selected *ucm* genes in *S. uncialis* strains following 5 days growth in different liquid media: (I) wild-type in ISP-4; (II) wild-type in ISP-4M; and (III) SB18002 (Δ*clao-D* mutant) in ISP-4M. The *rpoB* gene was served as a positive control for both the RT-PCR and overall RNA level. (C) Metabolite profiles of *S. uncialis* strains upon HPLC analysis with UV detection at 540 nm following 7 days fermentation in various media: (I) wild-type on ISP-4 agar; (II) wild-type in ISP-4 liquid; (III) wild-type in ISP-4M liquid; (IV) SB18002 (Δ*claO-D*) in ISP-4M liquid; and (V) SB18013 (Δ*claO-D/*Δ*ame3–6*) in ISP-4M. UCM, (○); CLA A, (◆); other CLAs, (◊); AMEs, (∇). See Supplementary Fig. S4 for metabolite profiles at 254 nm.

Strain improvement of the TNM A producer *Streptomyces* sp. CB03234, in combination with medium optimization, has successfully improved TNM A production to exceed 20 mg/l (Liu et al., 2018, 2020; Zhuang et al., 2019). Subsequently, the engineered *S.* sp. CB03234 strains have also been developed as a biotechnology platform for the production of other members of the anthraquinone-fused subfamily of enediynes and their engineered analogues (Yan et al., 2018). Characterization of these new TNM congeners provided detailed insight into the late-stage biosynthesis of TNM A. It has also inspired the development of the TnmH methyltransferase-mediated biocatalytic strategy for preparation of the first generation of antibody−TNM conjugates (Adhikari, Teijaro, Yan, et al., 2020). The *ucm* BGC has been cloned from *S. uncialis* DCA2648 previously (Fig. 2A) (Yan et al., 2016). However, the inability of *S. uncialis* DCA2648 to produce UCM in submerged fermentation has prevented us from applying similar strain improvement and medium optimization strategies to improve UCM production and enable UCM biosynthetic and engineering studies.

Here, we report (i) development of a submerged fermentation condition for UCM production; (ii) deletion of BGCs encoding unrelated major metabolites to facilitate UCM isolation; (iii) improvement of UCM titers to ∼1 mg/l through sequential chemical mutagenesis, manipulation of pathway-specific regulators, and medium optimization; and (iv) *in vivo* inactivation of selected genes within the *ucm* BGC to demonstrate the feasibility for UCM biosynthesis and engineering in the newly generated *S. uncialis* strains. These findings should greatly facilitate the fundamental studies on UCM biosynthesis and engineering and accelerate the development of UCM and designer analogues as promising payload candidates for the next generation of anticancer ADCs.

## Materials and Methods

### Bacterial Strains, Plasmids, and Chemicals

Bacterial strains, plasmids/cosmids, and oligonucleotides used in this study are listed (Supplementary Tables S1 and S2). The cosmid library of *S. uncialis* was reported previously (Yan et al., 2016). PCR primers were purchased from Sigma-Aldrich. Commercial kits (Omega Bio-Tek) were used for gel extraction and plasmid preparation. All restriction endonucleases, Q5^®^ high fidelity, OneTaq^®^ DNA polymerase, and T4 DNA ligase were purchased from NEB and the reactions were performed according to the manufacturer's procedures. DIG High Prime DNA Labeling and Detection Starter Kit I (Roche) was used for Southern blot analysis. Other common chemicals and culture media components were purchased from standard commercial sources and used without any further modification.

### General Experimental Procedures

*Escherichia coli* strains harboring plasmids or cosmids were grown in lysogeny broth (LB) with appropriate antibiotics, at concentrations of ampicillin (100 μg/ml), apramycin (50 μg/ml), chloramphenicol (25 μg/ml), kanamycin (50 μg/ml), nalidixic acid (25 μg/ml), and thiostrepton (30 μg/ml) (Sambrook & Russel 2001). The non-methylating *E. coli* ET12567/pUZ8002 was used for intergeneric conjugation with *Streptomyces* strains, and the conjugations were carried out following standard procedures (Kieser et al., 2000). The wild-type and mutant strains of *S. uncialis* DCA2648 were cultivated at 28°C on solid ISP-4 medium (Shirling & Gottlieb 1966) for sporulation and in liquid tryptic soy broth (TSB) to prepare the mycelium for genomic DNA (gDNA) isolation. Isolation of gDNA from *Streptomyces* strains were performed using the salting out protocol (Kieser et al., 2000). Fermentation was carried out in New Brunswick Scientific Innova 44 incubator shakers. Analytical and preparative HPLCs were carried out on an Agilent 1260 Infinity LC System with C18 columns (4.6 × 250 mm, 5 μm, and 21.2 × 250 mm, 5 μm, respectively). HPLC-MS was conducted using an Agilent 1260 Infinity LC coupled to a 6230 TOF (HR-ESI) equipped with an Agilent Poroshell 120 EC-C18 column (4.6 × 50 mm, 2.7 μm). All ^1^H and ^13^C NMR spectra were collected with a Bruker Avance III Ultrashield 700 at 700 MHz for ^1^H and 175 MHz for ^13^C nuclei. DNA amplification was conducted using a thermocycler (Bio-Rad).

### Fermentation, HPLC Analysis, and TNM B Isolation

To produce UCM on ISP-4 solid agar, the *S. uncialis* DCA2648 wild-type was cultured following previously published procedure (Davies et al., 2005). Briefly, the ISP-4 solid agar culture (50 ml) of *S. uncialis* DCA2648 was grown for 14–21 days at 28°C and then extracted repeatedly with equal volume of ethyl acetate. The ethyl acetate extract was evaporated to dryness under reduced pressure. The resulting extracts were dissolved in 240 µl of methanol, 10 μl of which was subjected to HPLC or HPLC-MS analyses under UV detection at 540 nm.

To produce UCM by submerged fermentation, the *S. uncialis* DCA2648 wild-type and engineered strains were cultured in 250-ml baffled flasks containing 50 ml of the MYM medium (maltose 4 g/l, yeast extract 4 g/l, malt extract 10 g/l, pH 7.3) as seed cultures. After growth at 28°C and 250 rpm for 2 days, 5 ml of the seed cultures were inoculated into 250 ml baffled flasks containing 50 ml of various production media (Supplementary Table S3). The cultivation was continued at 28°C and 250 rpm for 7 days. The fermentation cultures (50 ml) of *S. uncialis* strains were centrifuged, yielding supernatant and biomass fractions. Supernatant was extracted with equal volume of ethyl acetate, and biomass was extracted with acetone. Both extracts were combined and evaporated to dryness under reduced pressure. The resulting residues were dissolved in 240 μl of methanol, 10 μl of which was subjected to HPLC or HPLC-MS analyses under UV detection at 540 nm. Analytical HPLC was carried out using a 15-min solvent gradient, from 90% solvent A (0.1% HOAc) and 10% solvent B (CH_3_CN) to 30% A and 70% B over 5 min, an isocratic step using 30% A and 70% B for 5 min, a gradient from 70% B to 100% B over 2 min, and a final washing step of 100% B for 3 min. HR-ESI-MS analysis (negative ion mode) was conducted using the same 15-min solvent gradient.

For large-scale fermentation and isolation of TNM B, spores of *S. uncialis* SB18012 were cultured in 250 ml baffled flasks containing 50 ml of the MYM seed medium. After growth at 28°C and 250 rpm for 2 days, 40 ml of the seed cultures were inoculated into 2-l flasks containing 400 ml of the optimized production medium (Supplementary Table S3) and incubated at 28°C and 250 rpm for 7 days. In total, 10-l cultures were extracted as described above. The ethyl acetate and acetone extracts were combined and evaporated to dryness, and the residues were dissolved in minimum methanol and fractionated by Sephadex LH-20 chromatography using methanol as elution solvent. Six fractions were obtained, and TNM B was enriched in one fraction as monitored by HPLC. Finally, 0.2 mg of TNM B was purified from this fraction by using a prep-HPLC and following a previous procedure (Yan et al., 2016).

### Transcriptional Analysis of the *ucm* Biosynthetic Genes

Total RNAs were isolated from *S. uncialis* cells (∼100 mg) using the SV total RNA isolation system (Promega) with minor modifications. Cell lysates were extracted with chloroform, prior to RNA purification using spin column (Takara). RNA samples were treated with DNAse I (ThermoFisher), followed by quantification using a Nanodrop spectrophotometer. Semi-quantitative RT-PCR was conducted as described previously (Hindra et al., 2010), using the OneStep RT-PCR kit (Qiagen) and the primers listed (Supplementary Table S2). The housekeeping gene *rpoB* (RNA polymerase β subunit-encoding gene) was used as an internal control for RNA levels and RNA integrity. The number of PCR cycles used for amplifying *rpoB* and *ucm* gene cDNAs was 25 and 30, respectively, with 32 cycles used for *ucmE10.* To ensure that any amplification observed was not due to residual DNA in RNA samples or other DNA contamination, RNA samples (without reverse transcription) were checked by PCR. PCR products were separated by electrophoresis on agarose gels with GelRed precast staining (Biotium). Transcriptional analyses were conducted using at least two independent RNA samples.

### Construction of the Δ*claO-D* and *Δame3-6* Mutants *S. uncialis* SB18002 and SB18013

To generate the Δ*claO-D* in-frame mutant in *S. uncialis* DCA2648, the cosmid construct pBS18008 was used. It spans loci AB852_16715 to AB852_16630 (GenBank accession no. LFBV01000003), including the whole cladoniamide (CLA) BGC (Ryan, 2011). In *E. coli* BW25113/pIJ790, neomycin resistance cassette of cosmid pBS18008 was replaced with an apramycin resistance cassette as *S. uncialis* is naturally resistant to kanamycin, to give pBS18009. The gene locus harboring *claO-D* genes (Supplementary Fig. S5) was replaced with a neomycin + FLP cassette (amplified using oligonucleotides KO*claO*-f/KO*claD*-r, Supplementary Table S2), to give pBS18010 (Gust et al., 2003). The neomycin cassette was flipped out in *E. coli* DH5α/BT340, yielding pBS18011. This construct was passaged to *E. coli* strain ET12567/pUZ8002 and subsequently introduced into *S. uncialis* DCA2648. The Δ*claO-D* in-frame mutant was identified by the single crossover-apramycin resistant selection, followed by the double crossover-apramycin sensitive screening, and verified by PCR using oligonucleotides *claY*-S/*claD*-AS (Supplementary Table S2). The genotype of the Δ*claO-D* in-frame mutant, *S. uncialis* SB18002, were confirmed by Southern blot analysis (Supplementary Fig. S5). DNA probe was PCR-amplified using oligonucleotides *claX2*-Sin/*claX2*-ASin (Supplementary Table S2) and *S. uncialis* DCA2648 genomic DNA as template.

To generate the Δ*ame3–6* in-frame mutant in *S. uncialis* SB18002, the cosmid construct pBS18020 was used, which included *ame* BGC (Luo et al., 2021). In *E. coli* BW25113/pIJ790, neomycin resistance cassette of cosmid pBS18020 was replaced with an apramycin resistance cassette, to give pBS18021. The gene locus harboring *ame3–6* genes (Supplementary Fig. S5) was replaced with a neomycin + FLP cassette, to give pBS18022. The neomycin cassette was flipped out in *E. coli* DH5α/BT340, yielding pBS18023. This construct was passaged to the non-methylating *E. coli* strain ET12567/pUZ8002 and subsequently introduced into *S. uncialis* SB18002. The Δ*ame3–6* in-frame mutant *S. uncialis* SB18013 was identified by the single crossover-apramycin resistant selection, followed by the double crossover-apramycin sensitive screening, and verified by PCR using oligonucleotides *ame36-*S*/ame36-*AS (Supplementary Table S2 and Fig. S5).

### Isolation of UCM Producers *S. uncialis* SB18004 and SB18005 by Random Mutagenesis

Random mutagenesis of *S. uncialis* SB18002 was carried out to isolate UCM producers with higher titers (Supplementary Fig. S6). A total of 2700 colonies were selected after treatment of spore suspension with diethyl sulfate (0.1 ml diethyl sulfate in 0.3 ml EtOH added to 5 ml spore suspension, shake 45 min, survival rate 0.4%) (Chen et al., 2009). Mutants were bio-assayed using a paper disc-agar diffusion method using *Micrococcus luteus* ATCC 9431 as the indicator (Chen, Yin, et al., 2010; Xu et al., 2005). Briefly, each colony after mutagenesis was transferred into duplicate ISP-4 plates and incubated at 28°C for 10–14 days. Then, colonies from one of the duplicate plates were plugged out and transferred into a 1.5-ml Eppendorf tube in which 0.5 ml of methanol was added. After shaking for 6 hr, methanol extracts were centrifuged for 15 min at 5000 g, and 10 µl of the extract was transferred onto each paper disc in comparison with the parent *S. uncialis* SB18002 strain. Twenty isolates providing the largest inhibition zones were further validated for UCM production via submerged fermentation and HPLC analyses, leading to the identification of *S. uncialis* SB18004 and SB18005 as the two strains with the highest UCM titers.

### Generation of the *ucmR4R7*-Overexpressing Strains *S. uncialis* SB18006, SB18007, and SB18012

To construct the *ucmR4* and *ucmR7* overexpression plasmid pBS18013, both genes were PCR-amplified using *S. uncialis* DCA2648 genomic DNA as template and oligonucleotides *ucmR4*-S*Xba*I/*ucmR4*-AS*Sbf*I*Eco*RI and *ucmR7*-S*Sbf*I/*ucmR7*-AS*Eco*RI (Supplementary Table S2), respectively. Both *ucmR4* and *ucmR7* fragments were digested and simultaneously cloned into the backbone of pBS9083 (Hindra et al., 2017) that was previously digested by *Xba*I and *Eco*RI and purified by gel extraction, affording pBS18012. The *ucmR4R7* insert and the ErmE* promoter was extracted from pBS18012 by *Spe*I and subsequently inserted to pRT801AT using the same site, to give *ucmR4R7*-overexpressing vector pBS18013. This construct was introduced into *S. uncialis* SB18004, SB18005, and SB18008 through intergeneric conjugation as described above, respectively. The recombinant strains *S. uncialis* SB18006, SB18007, and SB18012, respectively, were obtained using thiostrepton selection, and verified by PCR using oligonucleotides P*ermE*-S/*ucmR4*-AS (Supplementary Table S2).

### Single Factor Medium Optimization of *S. uncialis* SB18007 for UCM Production

The first generation of liquid production medium (ISP-4M) is formulated on the basis of ISP-4 liquid medium supplement with 2.5 g/l malt extract, 10 mg/l CuSO_4_·5H_2_O, and 5 mg/l NaI (Supplementary Table S3). To formulate the second generation of production medium, the inorganic salts of ISP-4M medium were set as the constant components, and the effects of various nitrogen sources (malt extract, yeast extract, beef extract, corn steep solid, NZ-amine, peptone, and soytone) and carbon sources (soluble starch, dextrin, glucose, sucrose, mannitol, maltose, glycerol, galactose, fructose, and lactose) on UCM production by *S. uncialis* SB18007 were then evaluated (Supplementary Table S4).

### Inactivation of *ucmM* and *ucmP* in *S. uncialis* SB18002 and Complementation of the Resultant Δ*ucmM* and Δ*ucmP* Mutant Strains *S. uncialis* SB18008 and SB18009 in trans

The gene replacement method described above was used to generate the Δ*ucmM* and Δ*ucmP* mutants (Supplementary Fig. S7) (Gust et al., 2003). The *ucmM* or *ucmP* gene in cosmid pBS18003 was replaced with the apramycin resistance cassette to give pBS18014 or pBS18015, respectively. Then pBS18014 or pBS18015 were introduced into *S. unci*alis SB18002 by intergeneric conjugation, respectively. The double crossover mutants were selected using apramycin and further screened by PCR using oligonucleotides used in the gene complementation experiments (Supplementary Table S2 and Fig. S7). The genotype of the *S. uncialis* SB18008 (Δ*ucmM*) and SB18009 (Δ*ucmP*) mutants were confirmed by Southern blot analyses (Supplementary Fig. S7).

To determine that the Δ*ucmM* or Δ*ucmP* mutation affected only the targeted gene, DNA fragment of the *ucmM* or *ucmP* gene was amplified using *ucmM*-S*Xba*I2/*ucmM*-AS*Eco*RI2 or *ucmP*-S*Xba*I/*ucmP*-AS*Eco*RI (Supplementary Table S2) and cloned into the backbone of pBS9083, affording pBS18016 or pBS18017, respectively. The inserts, including the ErmE* promoter, were extracted from pBS18016 and pBS18017 by *Spe*I and cloned into pRT801AT at the *Spe*I site, to yield pBS18018 and pBS18019, respectively. The introduction of pBS18018 into *S. uncialis* SB18008 and pBS18019 into *S. uncialis* SB18009 were achieved by intergeneric conjugation described above, with selection for the thiostrepton-resistant phenotype, affording the complementation strains *S. uncialis* SB18010 (i.e., Δ*ucmM*/ErmE*-ucmM) and SB18011 (i.e., ucmP/ErmE*-ucmP), respectively, which were verified by PCRs using oligonucleotides 801-S/801-AS (Supplementary Table S2).

## Results and Discussion

### Activation of the *ucm* BGC During Submerged Fermentation of *S. uncialis* DCA2648

The first attempt of submerged fermentation of *S. uncialis* DCA2648 for UCM production was tested using ISP-4 liquid medium with solid agar as positive control, and UCM was not detected in the liquid fermentation culture (Fig. 2C, panels I and II). We reasoned that the absence of UCM production during submerged growth was due to lack of gene expression within the *ucm* BGC. To establish the transcription profile of the *ucm* BGC under ISP-4 solid and submerged fermentation conditions, RNA samples were prepared from *S. uncialis* DCA2648 cells grown on ISP-4 agar plate and ISP-4 liquid broth, followed by RT-PCR and electrophoretic analysis of the PCR products corresponding to each of the *ucm* genes. The transcription profile suggested that most of the *ucm* genes were transcribed after 14 days of growth on ISP-4 agar plate (Supplementary Fig. S2). In contrast, majority of the *ucm* genes were silent during submerged fermentation using ISP-4 liquid medium (Fig. 2B, panel I).

We noticed that the liquid media for DYN, TNM, and YPM production all contain copper sulfate (CuSO_4_·5H_2_O) and sodium iodide (NaI) (Supplementary Table S3) (Lam et al., 1992; Yan et al., 2017, 2018). It has been reported that supplementing the culture of *Micromonospora chersina*, the wild-type producer of DYN A, with 0.5 mg/l NaI can boost the yields of the DYN A by 300-fold (Cohen & Townsend, 2018), and an iodoanthracene has been identified as an on-pathway biosynthetic intermediate for the anthraquinone moiety of DYN A (Cohen & Townsend, 2018). These results suggested a specific role for iodide in the biosynthesis of anthraquinone-fused subfamily of enediynes. The DYN and TNM liquid production media were then tested for *S. uncialis* DCA2648 wild-type but again no UCM production was detected (Supplementary Fig. S3). To find a submerged cultivation condition where the *ucm* BGC is activated, we rationally modified the medium based on both the original ISP-4 agar medium and the reported liquid media for DYN, TNM, and YPM production. By supplementing ISP-4 liquid medium with malt extract, CuSO_4_·5H_2_O, and NaI (ISP-4M medium, Supplementary Table S3), the *ucm* BGC was activated as indicated by transcriptional analysis (Fig. 2B, panel II). The UCM production was confirmed by HPLC-MS analysis of fermentation broth using authentic UCM as a standard (Fig. 2C and Supplementary Fig. S4). Under this submerged fermentation condition, the titer of UCM (0.03 ± 0.02 mg/l) is comparable to that obtained by of solid-state fermentation.

### Elimination of Major Metabolites of *S. uncialis* DCA2648 to Facilitate UCM Detection and Isolation

Under both solid-state and the established submerged fermentation condition, HPLC analysis of *S. uncialis* DCA2648 wild-type fermentation revealed a major metabolite with an overlapping retention time (13.7 min) to UCM (Fig. 2C panel III and Supplementary Fig. S4A). HR-ESI-MS analysis of this metabolite yielded an [M–H]^–^ ion at *m/z* 436.0686 (Supplementary Fig. S4B), indicative of CLA A, a cladoniamide produced by *S. uncialis* DCA2648 with high titers (∼1.2 mg/l) (Williams et al., 2008). Under our submerged fermentation condition, CLAs were also produced as major metabolites as shown in the HPLC metabolite profile with detection at UV–Vis 254 nm (Supplementary Fig. S4A). To remove potential competition for nutrients and precursors and to simplify the detection for and isolation of the minor metabolite UCM, we decided to eliminate the production of CLAs by deleting the essential *cla* biosynthetic genes, which have been cloned (Ryan, 2011). By PCR-targeted gene replacement, we generated the Δ*claO-D* in-frame deletion mutant *S. uncialis* SB18002, the genotype of which was confirmed by Southern blot analysis (Supplementary Fig. S5). The expression of the *ucm* BGC was not affected by deletion of the *cla* genes (Fig. 2B, panel III). HPLC analysis of *S. uncialis* SB18002 fermentation, with the *S. uncialis* DCA2648 wild-type as a control, showed complete abolishment of CLAs production (Fig. 2C panel IV and Supplementary Fig. S4A). UCM titer in *S. uncialis* SB18002, however, remained at ∼0.03 mg/l, which was the same as that from the *S. uncialis* DCA2648 wild-type.

HPLC analysis of *S. uncialis* SB18002 submerged fermentation has also led us to discover and characterize another group of novel metabolites, the ammosesters (AMEs) (Luo et al., 2021). To further facilitate the UCM isolation process, we generated the Δ*ame3–6* in-frame deletion in the Δ*cla* mutant *S. uncialis* SB18002, by PCR-targeted gene replacement, to afford the Δ*cla*/Δ*ame* double-deletion mutant *S. uncialis* SB18013 (Supplementary Fig. S5). Though the metabolite profile of *S. uncialis* SB18013 fermentation is very clean in comparison with those from the *S. uncialis* DCA2648 wild-type or the Δ*cla* mutant SB18002, UCM titer, however, was not improved, prompting us to subject *S. uncialis* SB18002 to the subsequent strategies for further titer improvement (Fig. 2C, panel V).

### Multiple Strategies to Improve UCM Titers by Submerged Fermentation of *S. uncialis* Strains

**Fig. 3.**
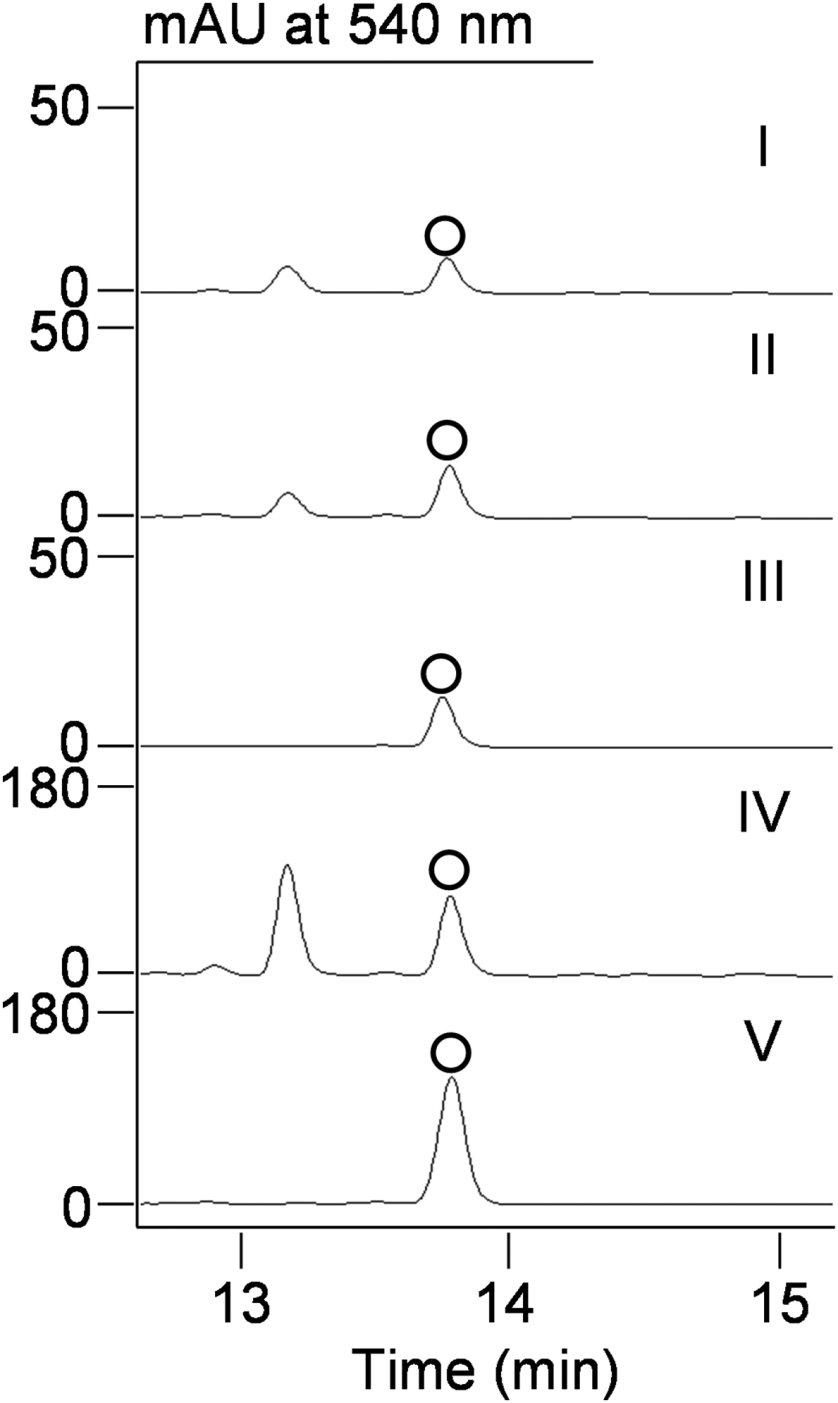
HPLC analysis of UCM (○) production in *S. uncialis* SB18002 and mutant strains following chemical mutagenesis and overexpression of pathway-specific activator genes: (I) SB18002; (II) SB18004; (III) SB18005; (IV) SB18006 (*ucmR4R7* overexpressing in SB18004); and (V) SB18007 (*ucmR4R7* overexpressing in SB18005).

We undertook multiple strategies to improve UCM production, including traditional strain improvement by chemical mutagenesis, manipulation of pathway-specific regulators, and medium optimization, all of which have been widely used to enhance the production titers of microbial metabolites (Adrio & Demain, 2006; Baltz, 2016; Chen, Smanski, et al., 2010; Demain, 2006; Teijaro et al., 2019). Since these experiments were performed before the construction of the Δ*cla*/Δ*ame* double-deletion mutant *S. uncialis* SB18013, the Δ*cla* mutant *S. uncialis* SB18002 was used instead. We first subjected *S. uncialis* SB18002 to diethyl sulfate, a DNA alkylating agent, for random mutagenesis using an established process for screening high producing strains (Supplementary Fig. S6) (Xu et al., 2005). Around 2700 individual colonies were selected and screened, using an antibacterial assay with *M. luteus* as the indicator strain, for methanol extracts with inhibition zones larger than *S. uncialis* SB18002. Twenty mutants generating the largest inhibition zones were further validated via submerged fermentation in shaking flasks. The two best mutants, named *S. uncialis* SB18004 and SB18005, produced UCM with titers at ∼0.07 ± 0.05 mg/l, representing at least twofold improvement over that obtained from the parent *S. uncialis* SB18002 (Fig. 3).

We further improved the UCM titer by overexpressing the pathway-specific positive regulators within the *ucm* BGC. Bioinformatics analysis of the *ucm* BGC allowed for the identification of two genes, *ucmR4* and *ucmR7*, that likely encode pathway-specific positive regulators. The encoded UcmR4 and UcmR7 are members of the AraC family of transcriptional regulators (Gallegos et al., 1997). UcmR7 homologues were conserved among several enediyne BGCs. UcmR7 is a homologue of SgcR2 from the 9-membered enediyne C-1027 biosynthetic machinery (34% identity/49% similarity), which has a positive regulatory role in C-1027 production (Chen, Yin, et al., 2010). Unlike *ucmR7, ucmR4* was specific to the BGCs of the anthraquinone-fused subfamily of enediynes (Yan et al., 2017). Transcriptional analysis of the *ucm* BGC revealed that *ucmR4* and *ucmR7* expression is positively correlated with the expression of other *ucm* genes during growth on ISP-4 agar plates (Supplementary Fig. S2), supporting UcmR7 and UcmR4 as transcriptional activators. We opted to construct a chromosomally integrated-overexpression plasmid pBS18013 harboring *ucmR4* and *ucmR7* in tandem under the control of the constitutive ErmE* promoter. The pBS18013 was then introduced into both *S. uncialis* SB18004 and SB18005 to give *S. uncialis* SB18006 and SB18007, respectively. The fermentations of *S. uncialis* SB18006 and SB18007, with *S. uncialis* SB18002 as a control, followed by HPLC analysis revealed that significant UCM titer improvement in both *S. uncialis* SB18006 and SB18007, with *S. uncialis* SB18007 reaching UCM titer at ∼0.3 ± 0.2 mg/l, which represents 4- or 10-fold improvement over that by *S. uncialis* SB18005 or SB18002, respectively (Fig. 3).

To explore the capacity of *S. uncialis* SB18007 for UCM production, the submerged production medium was reformulated based on the single factor optimization. Various nitrogen and carbon sources were evaluated. The evaluation results suggested that mannitol and malt extract were the best carbon and nitrogen source, respectively (Supplementary Table S4). Based on the above single-factor optimization experiments, the UCM production medium was then determined to consist of mannitol 10 g/l, malt extract 2.5 g/l, K_2_HPO_4_ 1 g/l, MgSO_4_·7H_2_O 1 g/l, NaCl 1 g/l, (NH_4_)_2_SO_4_ 2 g/l, FeSO_4_·7H_2_O 1 mg/l, MnCl_2_·4H_2_O 1 mg/l, ZnSO_4_·7 H_2_O 1 mg/l, CaCO_3_ 2 g/l, CuSO_4_·5H_2_O 0.01 g/l, and NaI 5 mg/l at pH 7.2. The highest UCM titer of 1.1 ± 0.3 mg/l was achieved in shaking flasks using the optimal fermentation medium, which represented over 50-fold improvement over the original titer reported for the *S. uncialis* DCA2648 wild-type fermented on solid agar medium (Davies et al., 2005). With the establishment of submerged fermentation, further titer improvement could now be performed by multi-factorial optimization experiments, testing multiple concentrations of medium components and controlling pH and dissolved oxygen, in fed-batch reactors to ultimately achieve industrial scale production.

### Inactivation of *ucmM* and *ucmP* in *S. uncialis* SB18002 Revealing New Insight into UCM Biosynthesis

**Fig. 4.**
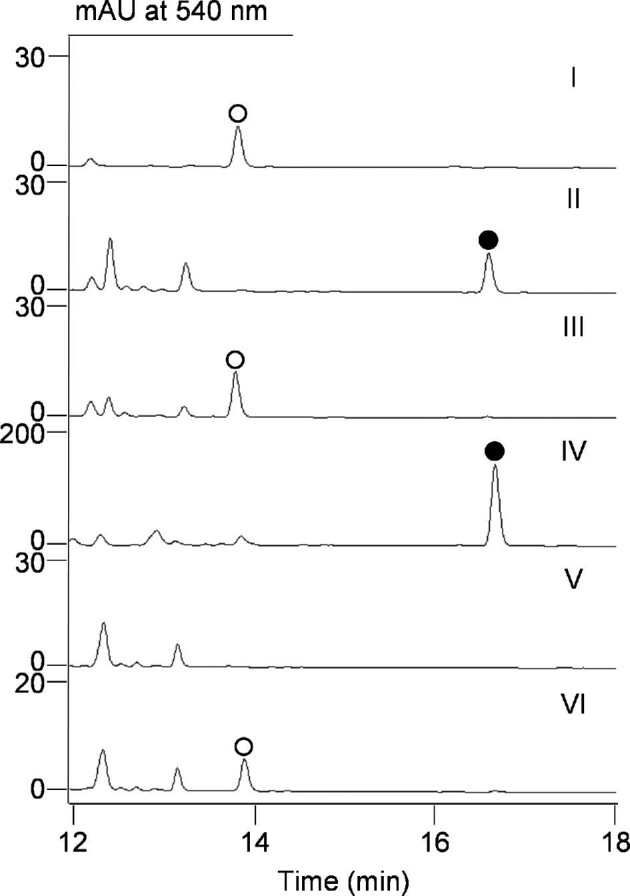
HPLC chromatograms of metabolite profiles from *S. uncialis* mutant strains: (I) SB18002; (II) SB18008 (Δ*ucmM)*; (III) SB18010 (Δ*ucmM + ucmM)*; (IV) SB18012 (Δ*ucmM* + *ucmR4R7* overexpression); (V) SB18009 (Δ*ucmP)*; and (VI) SB18011 (Δ*ucmP + ucmP)*. UCM, (○); TNM B, (●).

To demonstrate the feasibility of manipulating and studying UCM biosynthesis in *S. uncialis* two *ucm* genes were selected for inactivation *in vivo*. Bioinformatics analysis of the *ucm* BGC predicted *ucmP* and *ucmM* to encode a FAD-dependent oxidoreductase and a Rieske oxygenase, respectively. Previously, a genome neighborhood network analysis of the *dyn, tnm, ucm*, and *ypm* BGCs has suggested that *ucmP* is conserved among all four BGCs, while *ucmM* is conserved only among the *ucm, tnm*, and *ypm* BGCs but not the *dyn* BGC (Supplementary Fig. S1) (Yan et al., 2017). Thus, PCR-targeted replacement of *ucmM* and *ucmP* with an apramycin resistance cassette in *S. uncialis* SB18002 afforded the Δ*ucmM* and Δ*ucmP* mutants *S. uncialis* SB18008 and SB18009, respectively, the genotypes of which were confirmed by Southern analysis (Supplementary Fig. S7). Inactivation of *ucmM* or *ucmP* resulted in complete abolishment of UCM production as judged by HPLC-MS analysis (Fig. 4, panels II and V). UCM production can be readily restored to *S. uncialis* SB18010 or SB18011 upon expression of a functional copy of *ucmM* or *ucmP* in trans under the control of the ErmE* promoter (Fig. 4, panels III and VI). Taken together, these results conclusively established the essential roles of both *ucmM* and *ucmP* in UCM biosynthesis, demonstrating the feasibility of manipulating the UCM biosynthetic machinery *in vivo* under the submerged fermentation condition.

HPLC analysis of *S. uncialis* SB18008 fermentation also showed the accumulation of a new metabolite (Fig. 4, panel II). The fact that UCM production was fully restored to *S. uncialis* SB18008 upon expression of a functional copy of *ucmM* in trans would suggest the intermediacy of this new compound in UCM biosynthesis (Fig. 4, panel III). The titer of this new compound, however, was also low in *S. uncialis* SB18008. To facilitate its isolation, the *ucmR4R7* overexpression construct was introduced into *S. uncialis* SB18008 to afford *S. uncialis* SB18012, fermentation of which under the optimal condition yielded the new metabolite with a titer at ∼0.2 ± 0.1 mg/l (Fig. 4, panel IV). We subsequently scaled up *S. uncialis* SB18012 fermentation, and crude extracts prepared from 10-l of cultures were fractionated to yield 0.2 mg pure compound as a magenta oil. The molecular formula was assigned based on HR-ESI-MS analysis, affording a [M−H]^–^ ion at *m/z* 492.1094 (calculated [M−H]^–^ ion for C_29_H_18_NO_7_ at *m/z* 492.1088) (Supplementary Fig. S8). The structure was finally established on the basis of NMR analysis (Supplementary Fig. S9), as well as in comparison with ^1^H and ^13^C NMR spectra of UCM, yielding an identical structure as TNM B, an intermediate isolated previously from the Δ*tnmL* mutant *S*. sp. SB20020 for TNM A biosynthesis (Yan et al., 2018). Thus, the biosynthetic pathways of UCM and TNM shared a common intermediate TNM B, a finding that further supported the unified pathway for the biosynthesis of all members of the anthraquinone-fused subfamily of enediynes (Yan et al., 2018).

In summary, we have identified optimized cultivation media for the submerged fermentation of engineered *S. uncialis* strains to facilitate UCM production and isolation, with UCM titers reaching 1.1 mg/l, a near 58-fold increase over the original solid-state fermentation of the *S. uncialis* DCA2648 wild-type. The UCM overproducers were engineered by deletion of BGCs encoding unrelated major metabolites to simplify UCM detection and isolation, and random mutagenesis and overexpression of pathway-specific transcription activators to improve UCM titers. The characterization of TNM B from the Δ*ucmM* mutants *S. uncialis* SB18008 and SB18012 has provided new insight into UCM biosynthesis, further supporting the unified biosynthetic pathway for all members of the anthraquinone-fused subfamily of enediynes. Taken together, realization of submerged fermentation for the engineered *S. uncialis* strains with improved UCM titers and demonstration of the feasibility of manipulating the UCM biosynthetic machinery *in vivo* should inspire future efforts to further improve UCM production for ADC development by microbial fermentation, as well as biosynthetic and engineering studies for the anthraquinone-fused family of enediynes in general.

## Supplementary Material

kuab025_Supplemental_File

## Data Availability

All data generated or analysed during this study are included in this published article and its supplementary material.

## Supplementary Material

The strains, plasmids, primers, and media used in this study (Supplementary Tables S1–S3); effect of nitrogen and carbon sources on the production of UCM (Supplementary Table S4); genetic organization of *dyn, tnm, ucm*, and *ypm* BGCs (Supplementary Fig. S1); expression profiles for the *ucm* genes during growth on ISP-4 agar medium (Supplementary Fig. S2); UCM production by fermenting *S. uncialis* DCA2648 in DYN and TNM production media in comparison with ISP-4M (Supplementary Fig. S3); metabolite profiles of *S. uncialis* strains in various media (Supplementary Fig. S4); generation of mutants *S. uncialis* SB18002, SB18013, SB18008, and SB18009 (Supplementary Figs. S5 and S7); titer improvement of UCM production by chemical mutagenesis of *S. uncialis* SB18002 (Supplementary Fig. S6); HR-ESI-MS and NMR spectra of TNM B (Supplementary Fig. S8 and S9).
