# Supplementary material for: Submerged fermentation of Streptomyces uncialis providing a biotechnology platform for uncialamycin biosynthesis, engineering, and production
Source: J Ind Microbiol Biotechnol. 2021 Mar 19;48(3-4):kuab025. doi: 10.1093/jimb/kuab025 (PMC8210685; doi:10.1093/jimb/kuab025)
Supplement: kuab025_Supplemental_File [file kuab025_Supplemental_File.pdf]

Submerged fermentation of *Streptomyces uncialis* providing a biotechnology platform for uncialamycin biosynthesis, engineering, and production

Hindra,[1,4] Dong Yang,[1,3,4] Jun Luo,[1] Tingting Huang,[1] Xiaohui Yan,[1] Ajeeth Adhikari,[1] Christiana N. Teijaro,[1] Huiming Ge,[1] Ben Shen*,[1,2,3]

[1]Department of Chemistry, The Scripps Research Institute, Jupiter, Florida 33458, USA
[2]Department of Molecular Medicine, The Scripps Research Institute, Jupiter, Florida 33458, USA
[3]Natural Products Discovery Center at Scripps Research, The Scripps Research Institute, Jupiter, Florida 33458, USA

[4]These authors contributed equally
*Correspondence to: E-mail: shenb@scripps.edu; Tel: (561) 228-2456; Fax: (561) 228-2472

Supplementary Information (SI)

Table S1 Bacterial strains and plasmids used in this study S2
Table S2 Oligonucleotides used in this study S4
Table S3 Media used in this study S6
Table S4 Effect of nitrogen and carbon sources on the production of UCM S7
Figure S1 Genetic organization of *dyn*, *tnm*, *ucm*, and *ypm* biosynthetic gene clusters S8
Figure S2 Expression profiles for *ucm* genes in *S. uncialis* DCA2648 wild-type following growth on ISP-4 agar plates by RT-PCR analysis S9
Figure S3 Submerged fermentation of *S. uncialis* DCA2648 wild-type in DYN and TNM production media in comparison with the ISP-4M for UCM production S10
Figure S4 Metabolite profiles of *S. uncialis* strains following growth in various media S11
Figure S5 Generation of the Δ*claO-D* mutant *S. uncialis* SB18002 and the Δ*claO-D*/Δ*ame3-6* mutant *S. uncialis* SB18013 .... S12
Figure S6 Titer improvement of UCM production by chemical mutagenesis of *S. uncialis* SB18002 .... S13
Figure S7 Inactivation of *ucmM* and Δ*ucmP* by gene replacement in *S. uncialis* SB18002 .... S14
Figure S8 HR-ESI-MS spectrum of TNM B S15
Figure S9 ^{1}H and ^{13}C NMR spectra of TNM B in acetone-d_6 .... S16
Supplementary References S17

Table S1. Bacterial strains and plasmids used in this study

Strain	Description	Reference/Source
E. coli		
DH5α	*E. coli* host for plasmid construction	Life Technologies
ET12567/pUZ8002	Methylation-deficient *E. coli* host for intergeneric conjugation; contains pUZ8002, a non-transmissible *oriT* mobilizing plasmid	MacNeil et al., 1992
BW25113/pIJ790	*E. coli* host for PCR targeting	Gust et al., 2003
DH5α/BT340	*E. coli* host for excising resistance cassette	Gust et al., 2003
Micrococcus luteus ATCC 9431	indicator strain for bioassay of UCM production	Chen et al., 2010
S. uncialis		
DCA2648	wild-type	Davies et al., 2005
SB18002	DCA2648 *ΔclaO-D::*scar	This study
SB18004	SB18002 random mutant D1-1032	This study
SB18005	SB18002 random mutant D1-1329	This study
SB18006	pBS18013 integrated into SB18004, *ucmR4R7* overexpressing under *ErmE**	This study
SB18007	pBS18013 integrated into SB18005, *ucmR4R7* overexpressing under *ErmE**	This study
SB18008	SB18004 *ΔucmM::aac(3)IV*	This study
SB18009	SB18004 *ΔucmP::aac(3)IV*	This study
SB18010	pBS18018 integrated into SB18008, *ucmM* complementation	This study
SB18011	pBS18019 integrated into SB18009, *ucmP* complementation	This study
SB18012	pBS18013 integrated into SB18008, *ucmR4R7* overexpressing	This study
SB18013	SB18002 *Δame3-6::*scar	This study

Plasmid	Description	Reference/Source
pRT801	φBT1-based integrative cloning vector	Gregory et al., 2003
pRT801AT	pRT801-based vector, *aac(3)IV* cassette was replaced with ampicillin-thiostrepton tandem cassette	This study
pBS9083	Plasmid containing *zbmLG* with *ErmE** promoter	Hindra et al., 2017
pBS18003	Cosmid 20A3 containing partial *ucm* gene cluster	Yan et al., 2016
pBS18008	Cosmid 21E7 containing entire *cla* gene cluster	This study
pBS18009	Cosmid pBS18008 with *aac(3)IV*+oriT cassette replacing neomycin+FLP resistance in the backbone	This study
pBS18010	Cosmid pBS18009 containing *ΔclaO-D::neo*+FLP	This study
pBS18011	Cosmid pBS18009 containing *ΔclaO-D::scar*	This study
pBS18012	*ucmR4* and *ucmR7* cloned into pBS9083 backbone	This study
pBS18013	*ucmR4* and *ucmR7* with *ErmE** promoter cloned into pRT801AT	This study

Table S1 continued

Plasmid	Description	Reference/Source
pBS18014	Cosmid pBS18003 containing *ucmM::aac(3)IV*+oriT	This study
pBS18015	Cosmid pBS18003 containing *ucmP::aac(3)IV*+oriT	This study
pBS18016	*ucmM* cloned into pBS9083 backbone	This study
pBS18017	*ucmP* cloned into pBS9083 backbone	This study
pBS18018	*ucmM* with *ErmE** promoter cloned into pRT801AT	This study
pBS18019	*ucmP* with *ErmE** promoter cloned into pRT801AT	This study
pBS18020	Cosmid 13A9 containing *amm* gene cluster	This study
pBS18021	Cosmid pBS18020 with *aac(3)IV*+oriT cassette replacing neomycin+FLP resistance in the backbone	This study
pBS18022	Cosmid pBS18021 containing *Δame3-6::neo*+FLP	This study
pBS18023	Cosmid pBS18022 containing *Δame3-6::scar*	This study

Table S2. Oligonucleotides used in this study

Oligonucleotide	Nucleotide Sequence (5'-3')	Function/ reference
rpoB-RT	GATGTTGATCAGGGTCTGCG	Internal control for RT-PCR
rpoB-S	CATCGACCACTTCGGCAAC	Internal control for RT-PCR
ucmE3-S2	CCCTGGACGGCTACGGCTTC	RT-PCR
ucmE3-RT	CGGCGTGCGTCCGGGCCTCC	RT-PCR
ucmE4-S2	GGCTGCGTCCCGGGCGACT	RT-PCR
ucmtnmE4-RT	CGGGGAAGTAGTTGCCGACGA	RT-PCR
ucmE5-S2	CTTCGCCCTGTCGATCTCGGT	RT-PCR
ucmE5-RT2	GGTCGTTGGCGTAGAGCAGCAT	RT-PCR
PKSESUNC-S	TGCACTGGCTCGCGCTGGAC	RT-PCR
PKSESUNC-RT2	GCAGATCGAAGTGGTTGCAGA	RT-PCR
ucmT2-S	GCGTTCCCGCCGATGAGGAC	RT-PCR
ucmT2-RT	GCCGTCACCATCGACGTGATG	RT-PCR
ucmE10-S	CCACTACGTGTCCTGGCAGGG	RT-PCR
ucmE10-RT2	GCCGTCGCTGCGCATACAGG	RT-PCR
ucmtnmS3-S	CCAGGAGAAGGCCAAGGACTTC	RT-PCR
ucmS3-RT	GTTCGTCCGCACTCTGGTTGAG	RT-PCR
ucmR4-S	CACCACCCTGGCCGCCGAGG	RT-PCR
ucmtnmR4-RT2	CGGAGAACCGGGAGCTGAACG	RT-PCR
ucmtnmS2-S	TTCGAGGTCCGCAACGACATGA	RT-PCR
ucmtnmS2-RT	GAGGCTGAACCAGTTGCCGGA	RT-PCR
ucmtnmS1-S	GTCTTCGTCAACGACCAGGACA	RT-PCR
ucmtnmS1-RT	GGTCCTTGAAGCTGGCGTTGG	RT-PCR
ucmtnmT1-S	GTCGCCCTGGGCACGATGATG	RT-PCR
ucmtnmT1-RT	GAGGCCCGCCATCACCGAGG	RT-PCR
ucmR3-S2	GGCCGCCTGCTGTCCCTCAC	RT-PCR
ucmR3-RT2	GACAGGGCGCGGTCCAGTTG	RT-PCR
ucmC-S	CGGTGACCGTGGTGCACTGC	RT-PCR
ucmC-RT	GCTCCACAGCGACCACTCGG	RT-PCR
ucmD-S	CCCGGTACTGCTGGAGGACCT	RT-PCR
ucmtnmD-RT	GGTCAGCAGCTCGATCGCGTC	RT-PCR
ucmtnmF-S	GGCATCACCTCGGTCGACACC	RT-PCR
ucmF-RT2	GCGTAGGGGTCGAACGGGGT	RT-PCR
ucmN-S	GATCGACACCTGGAACAACG	RT-PCR, Southern analysis
ucmN-RT	AGATACAGTCCGTCGGCCA	RT-PCR, Southern analysis
ucmO-S	CCGTGGAGCCGTGCTTTTCAC	RT-PCR
ucmO-RT	CCCCCAGTGCTCGGCGATCC	RT-PCR, Southern analysis
ucmP-S	GCCGGACTCGCAACCGCCGT	RT-PCR
ucmP-RT	GTACAGGGCGTCGAGAAGATC	RT-PCR
ucmR2-S	CGCCGAGGCTTTGGTGACCCT	RT-PCR
ucmR2-RT	GTCGAGTCCGGCCAGGAACG	RT-PCR
ucmM-S	GGCGCGGACTTCCTGGGTGG	RT-PCR
ucmM-RT	CGCTCGCCGTTGAACGCCCA	RT-PCR
ucmtnmJ-S	GGCATGGTCAGCATCATGTG	RT-PCR
ucmJ-RT	TGCCACATCGTCCGGTACAG	RT-PCR
ucmK1-S	TGCCGGATCTGGTGCGGT	RT-PCR
ucmK1-RT	GCGCCCAACTGGTGGGAA	RT-PCR
ucmK2-S	GCGCCCAACTGGTGGGAA	RT-PCR
ucmK2-RT	GTTCCGCGAATTGGCGTAC	RT-PCR
ucmR1-S	GATCGTGCGGGAACTGCTCATC	RT-PCR
ucmtnmR1-RT	CCGTTGATCATCGCCTGGACG	RT-PCR
ucmtnmI-S	CCGGCACCACCGCCCTGGT	RT-PCR
ucmI-RT	AGCAGCAGGACACCGGCGTTG	RT-PCR
ucmB-S2	AGATTCCTCGGCGTCGGCAAC	RT-PCR

Table S2 continued

Oligonucleotide	Nucleotide Sequence (5'-3')	Function/ reference
ucmB-RT	GCGATGGGGAGCCAGTCG	RT-PCR
ucmR7-S	GAGGTCCAGTGCCTCTCCGA	RT-PCR
ucmR7-RT	CGGTCGCCGTGCTCGTCG	RT-PCR
ucmtnmG-S	GGCTGGTCAGCACCGTCACC	RT-PCR
ucmtnmG-RT	TTCTCGGCCTCCTCGACGACG	RT-PCR
KOclaO-f	GTGCGCAGGAACGGCTCGGCGGCCACGGG CGTCACCCGCATTCCGGGGATCCGTCGACC	Gene replacement
KOclaD-r	GAAGTCGCGGTGGAAGTCGTGGTGCTTCTC CAGGACGACTGTAGGCTGGAGCTGCTTC	Gene replacement
claY-S	TGACGGTCGGTGCTCCGGAC	Verification of mutant strain
claD-AS	GCTGAAGTCCGCGATGTCGATGC	Verification of mutant strain
claX2-Sin	CCCTCGACGTGGGCTTCTTCC	Southern analysis
claX2-ASin	GTCCTGAGCCGTTGAGGGTGG	Southern analysis
KOame3-f	AGAGGCCCTTTTTCGGCCCCCGCGTCAGAAA GCAGGTGCCATGATTCCGGGGATCCGTCGA	Gene replacement
KOame6-r	CGCCGAGCAGACCGGCCGCCGACGGGTCC GGCGACGGCCTCATGATGGCTGGAGCTGCT T	Gene replacement
ame36-S	CACACGATGGGGAGCGTCTGC	Verification of mutant strain
ame36-AS	CCGCAGCCCCGTGAACGAGG	Verification of mutant strain
ucmR4-SXbaI	ATATGTCTAGAGTGAGGAAGTGCACCCATTAA TG	Gene expression
ucmR4-ASSbfIEcoRI	GATAGGAATTCCCTGCAGGCTATCGAGGCGT CCGGTCAG	Gene expression
ucmR7-SSbfI	CAATCCTGCAGGACGAAGGGGACGACGGAC ATG	Gene expression
ucmR7-ASEcoRI	GATAGGAATTCGTCACCTGACGGCCCGGTTC	Gene expression
KOucmM-f	ATGACCGGGCTCGATCGTTCCGGCATTCCGT CCGCCGAGGAAATTCCGGGGATCCGTCGA	Gene replacement
KOucmM-r	TCAGGTCAGGAAGTCTCGTGCCGGGTTGGC CCGGGGGTAGCGTGTAGGCTGGAGCTGCTT	Gene replacement
KOucmP-f	GCAACCGCCGTCCTGCTGCGCCGCGCGGGT CTGGAGACCCGGATTCCGGGGATCCGTCGA	Gene replacement
KOucmP-r	GGCGGCGTCGAACGCGAACATGCCGCGCAA GCCCCGCAGTTGTGTAGGCTGGAGCTGCTT	Gene replacement
ucmO-Sin	CGGGAGGGTGTGGCGCA	Southern analysis
ucmM-SXbaI2	ATATGTCTAGACGCCTTATCGCGGCAGGG	Gene complementation and PCR verification
ucmM-ASEcoRI2	GATAGGAATTCGGTGCAAGCTGCCGCAC	Gene complementation and PCR verification
ucmP-SXbaI	ATATGTCTAGACACCATCGGCGGCTCCAACA C	Gene complementation and PCR verification
ucmP-ASEcoRI	GATAGGAATTCGCGAGGGTGAGGGAGCACA G	Gene complementation and PCR verification
PermE-S	GTGGCACCGCGATGCTGTTG	Verification of exconjugants
801-S	CTGTAGCGCACAGCGGGAGG	Verification of exconjugants
801-AS	CTTCCGGCTCGTATGTTGTGTG	Verification of exconjugants

Table S3. Media used in this study

Media	Composition	Reference
DYN	Soluble starch 10 g/L, pharmamedia 5 g/L, $CaCO_3$ 1 g/L, $CuSO_4{\bullet}5H_2O$ 0.05 g/L, NaI 5 mg/L.	Lam et al., 1992
TNM	Yeast extract 10 g/L, malt extract 10 g/L, maltose 10 g/L, $CuSO_4{\bullet}5H_2O$ 0.01 g/L, NaI 5 mg/L, pH 7.2	Yan et al., 2018
YPM	Maltose 10 g/L, yeast extract10 g/L, malt extract 10 g/L, $CaCO_3$ 2g/L, $CuSO_4{\cdot}5H_2O$ 0.01 g/L, NaI 5 mg/L, pH 7.2	Yan et al., 2017
ISP-4*	Soluble starch 10 g/L, K_2HPO_4 1g/L, $MgSO_4{\cdot}7\ H_2O$ 1 g/L, NaCl 1 g/L, $(NH_4)_2SO_4$ 2 g/L, $CaCO_3$ 2 g/L, $FeSO_4{\cdot}7H_2O$ 1 mg/L, $MnCl_2{\cdot}4H_2O$ 1 mg/L, $ZnSO_4{\cdot}7H_2O$ 1 mg/L, pH 7.2.	Shirling & Gottlieb 1966
ISP-4M	ISP-4 + malt extract 2.5 g/L, $CuSO_4{\bullet}5H_2O$ 0.01 g/L, NaI 5 mg/L, pH 7.2.	This study
Optimized UCM	Mannitol 10 g/L, malt extract 2.5 g/L, K_2HPO_4 1 g/L, $MgSO_4{\cdot}7H_2O$ 1 g/L, NaCl 1 g/L, $(NH_4)_2SO_4$ 2 g/L, $FeSO_4{\cdot}7H_2O$ 1 mg/L, $MnCl_2{\cdot}4H_2O$ 1 mg/L, $ZnSO_4{\cdot}7\ H_2O$ 1 mg/L, $CaCO_3$ 2 g/L, $CuSO_4{\cdot}5H_2O$ 0.01 g/L, NaI 5 mg/L, pH 7.2.	This study

*For solid media, add 20 g/L agar.

Table S4. Effect of nitrogen and carbon sources on the production of UCM in *S. uncialis* SB18007

Nitrogen sources (2.5 g/L)[a]	UCM (mg/L)[b]	Carbon sources (10 g/L)[c]	UCM (mg/L)[b]
Malt extract	0.3 ± 0.2	Soluble starch	0.3 ± 0.2
Beef extract	0.10 ± 0.05	Dextrin	0.8 ± 0.2
Corn steep solid	ND	Sucrose	0.5 ± 0.3
NZ-amine	ND	Mannitol	1.1 ± 0.3
peptone	0.10 ± 0.04	Galactose	ND
Soytone	ND	Fructose	ND
Yeast extract	ND	Glucose	0.2 ± 0.1
		Glycerol	ND
		Lactose	ND
		Maltose	ND

[a] The soluble starch and inorganic salts were set as the constant components.
[b] ND, not detected.
[c] The malt extract and inorganic salts were set as the constant components.

Figure S1. Genetic organization of *dyn*, *tnm*, *ucm* and *ypm* biosynthetic gene clusters. The two *ucm* genes inactivated in this study and their homologous genes (*ucmM/ypmM/tnmM1/M2* and *ucmP/ypmP/tnmP/dynE13,* respectively) are highlighted in red.

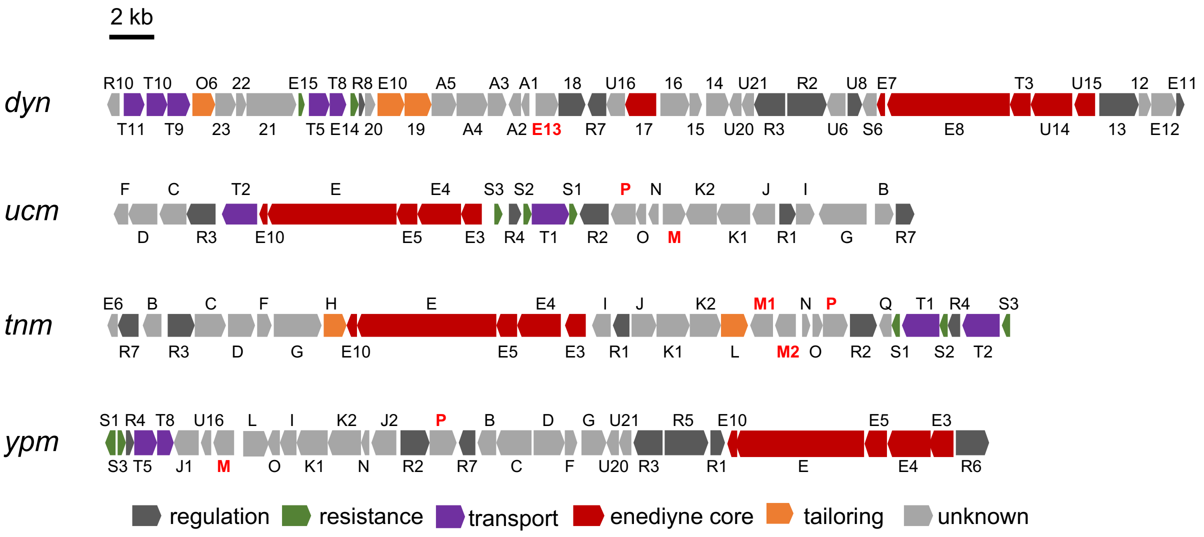

Figure S2. Expression profiles for *ucm* genes in *S. uncialis* DCA2648 wild-type following growth on ISP4 agar plates. The representative profiles were examined using semi-quantitative RT-PCR with 30 cycles of amplification, or 25 cycles for the RNA polymerase β subunit-encoding gene *rpoB* that served as a positive control for both the RT-PCR and for overall RNA level. These experiments were conducted using at least two independent RNA samples.

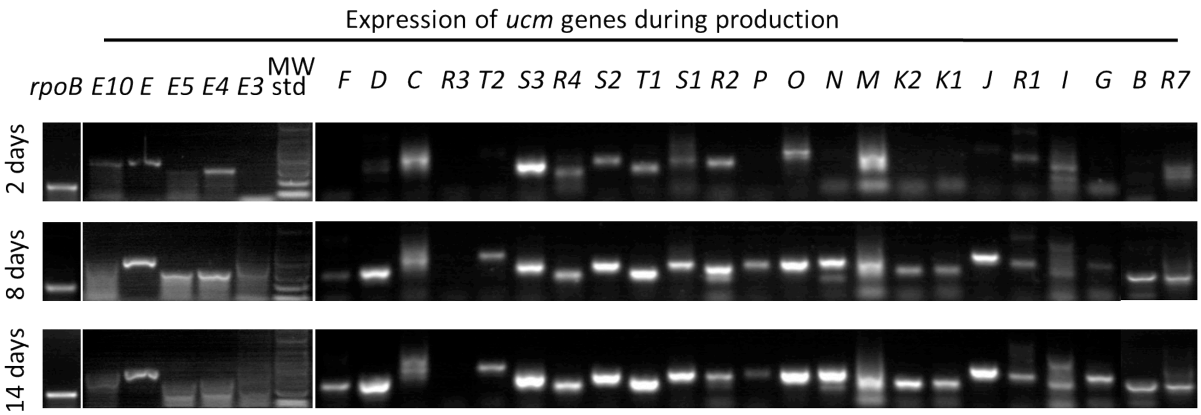

Figure S3. Submerged fermentation of *S. uncialis* DCA2648 wild-type in DYN and TNM production media in comparison with the ISP-4M for UCM production. (A) HPLC analyses of UCM production by submerged fermentation of *S. uncialis* DCA2648 wild-type in DYN and TNM production media in comparison with ISP-4M with UV detection at 540 nm. (B) The same HPLC analysis of UCM production with extracted ion chromatogram (EIC) of the UCM $[M-H]^-$ ion at *m/z* 438.09.

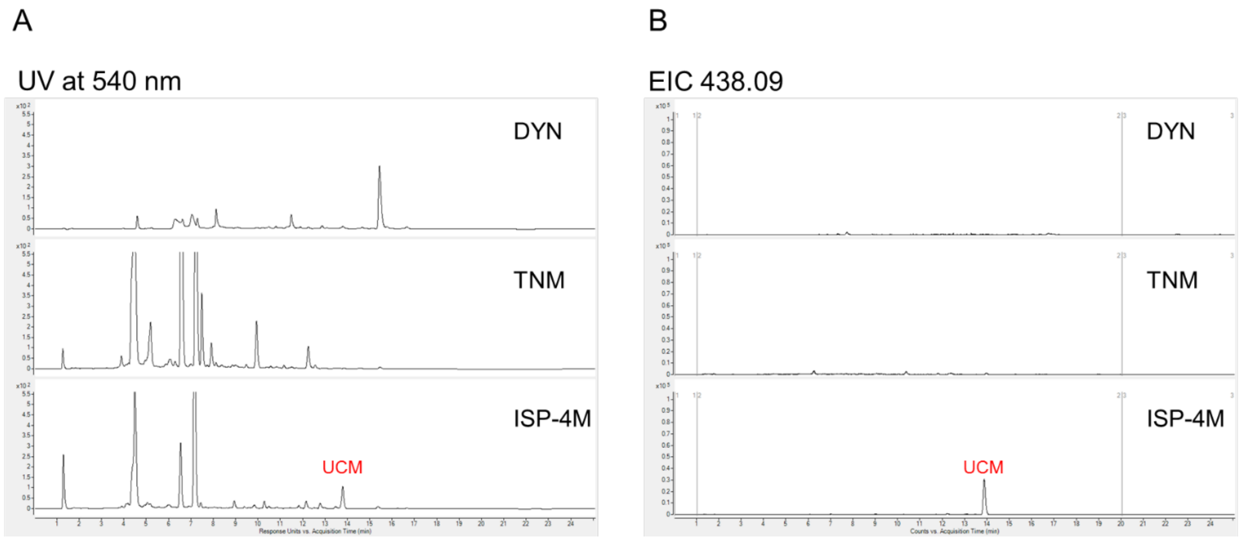

Figure S4. (A) Metabolite profiles of *S. uncialis* strains upon HPLC analysis with UV detection at 254 nm following seven days of fermentation in various media: (I) DCA2648 wild-type on ISP-4 agar; (II) DCA2648 wild-type in ISP-4 liquid; (III) DCA2648 wild-type in ISP-4M liquid; (IV) SB18002 (Δ*claO-D* mutant) in ISP-4M liquid; (V) SB18013 (Δ*claO-D*Δ*ame3-6* mutant) in ISP-4M liquid. UCM, (○); CLA A, (♦); other CLAs, (◊); AMEs, (∇). (B) HR-ESI-MS analysis for UCM and CLA A. UCM, calculated $[M – H]^–$ ion for $C_{26}H_{16}NO_6^–$ at *m/z* 438.0983 or calculated $[M + COOH]^–$ ion for $C_{27}H_{18}NO_8^–$ at *m/z* 484.1038; and CLA A, calculated $[M – H]^–$ ion for $C_{22}H_{15}{}^{35}ClN_3O_5^–$ at *m/z* 436.0706, and $[M – H]^–$ ion for $C_{22}H_{15}{}^{37}ClN_3O_5^–$ at *m/z* 438.0676, which is very close to $[M – H]^–$ ion of UCM.

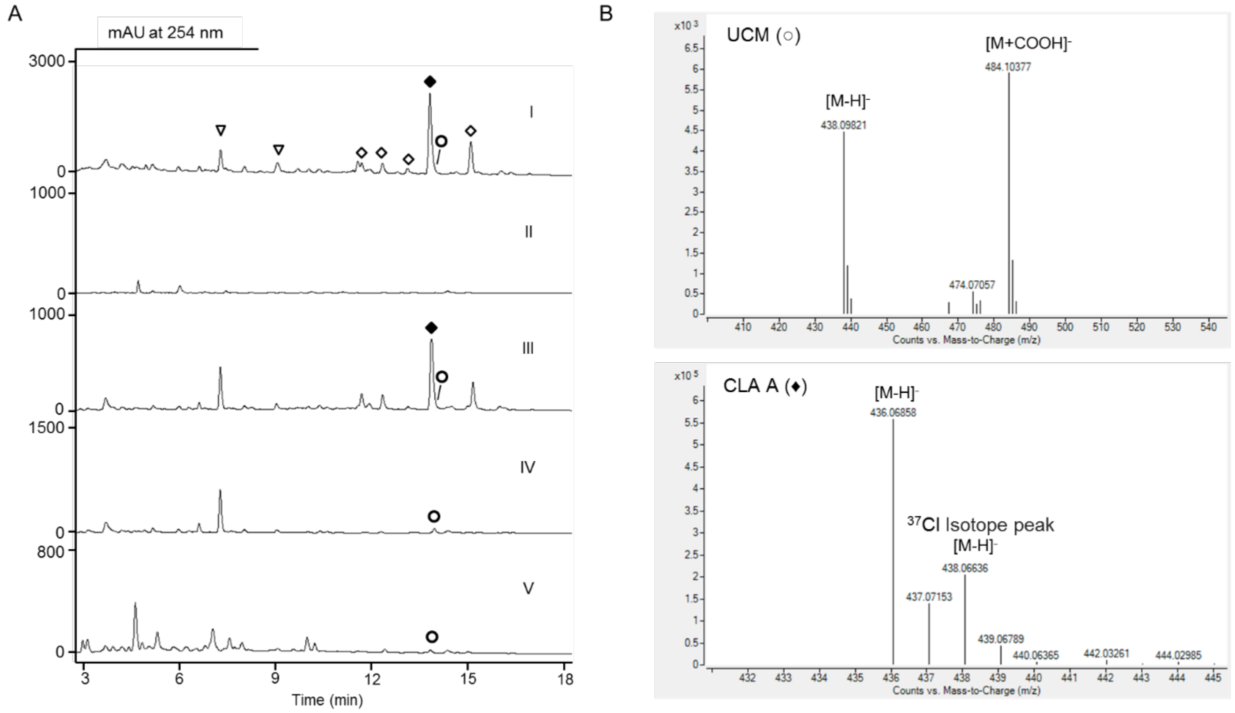

Figure S5. Generation of the Δ*claO-D* in-frame deletion mutant *S. uncialis* SB18002 and the Δ*claO-D*/Δ*ame3-6* double deletion mutant *S. uncialis* SB18013. (A) Schematic diagram of the gene replacement of *claO-D* (via homologous recombination) with a scar fragment containing 81-bp nucleotides designed for in-frame deletions without any stop codon. (B) Southern analysis of *S. uncialis* DCA2648 wild-type (lane 1) and SB18002 (lane 2) using *Bam*HI (Ba) digestion and DIG labelled-*claX2* PCR fragment (a 564-bp probe). Lane 3 represents molecular weight marker. (C) Schematic diagram of the gene replacement of *ame3-6* (via homologous recombination) with the scar fragment. (D) Confirmation of SB18013 by PCR using primers ame36-S/ame36-AS. Lane 1, SB18013; lane 2, SB18002 as negative control; lane 3, pBS18023 as positive control; lane 4, marker.

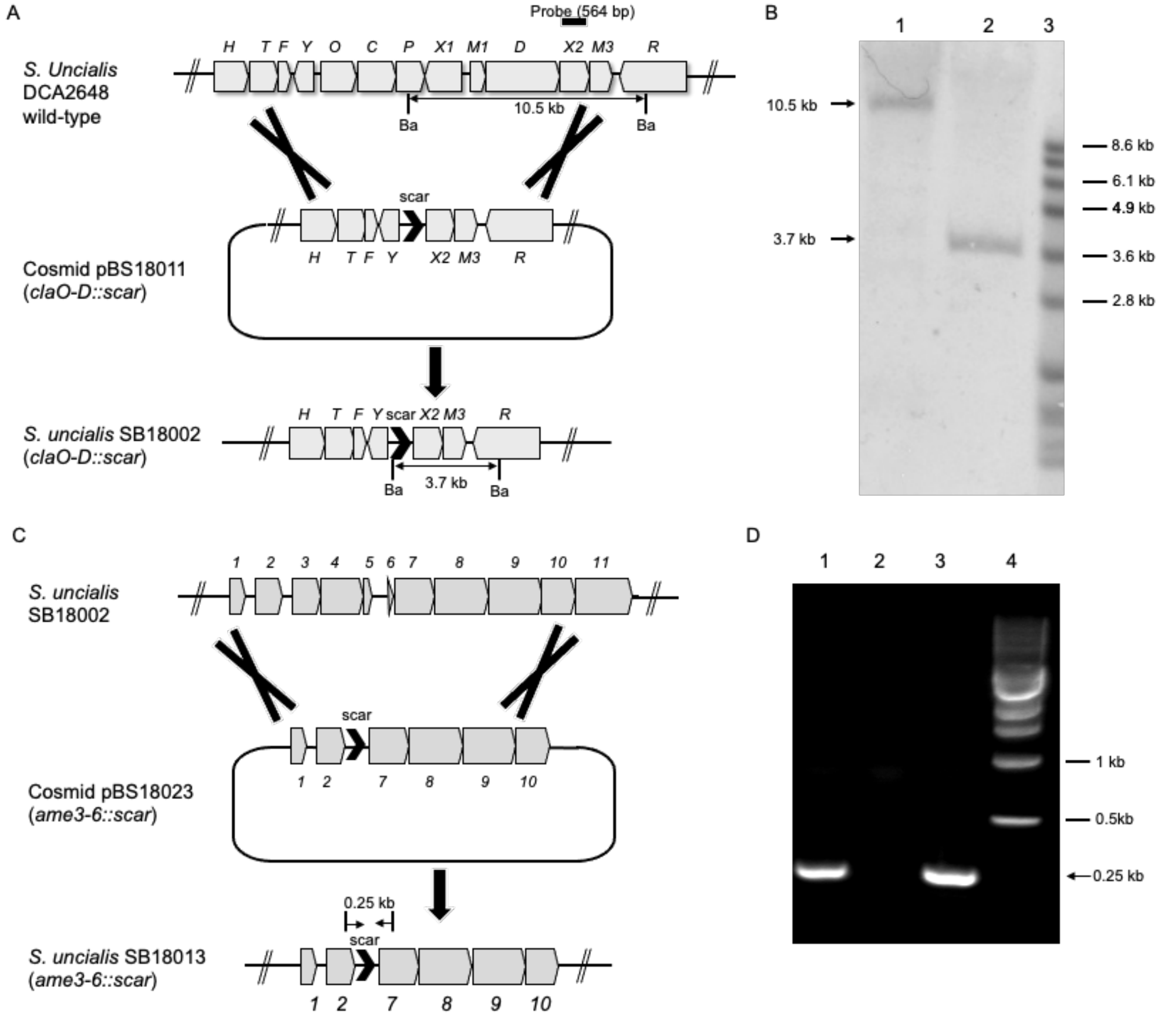

Figure S6. Titer improvement of UCM production by chemical mutagenesis of *S. uncialis* SB18002 using an integrated process with a modified agar plug method and *M. luteus* bioassay.

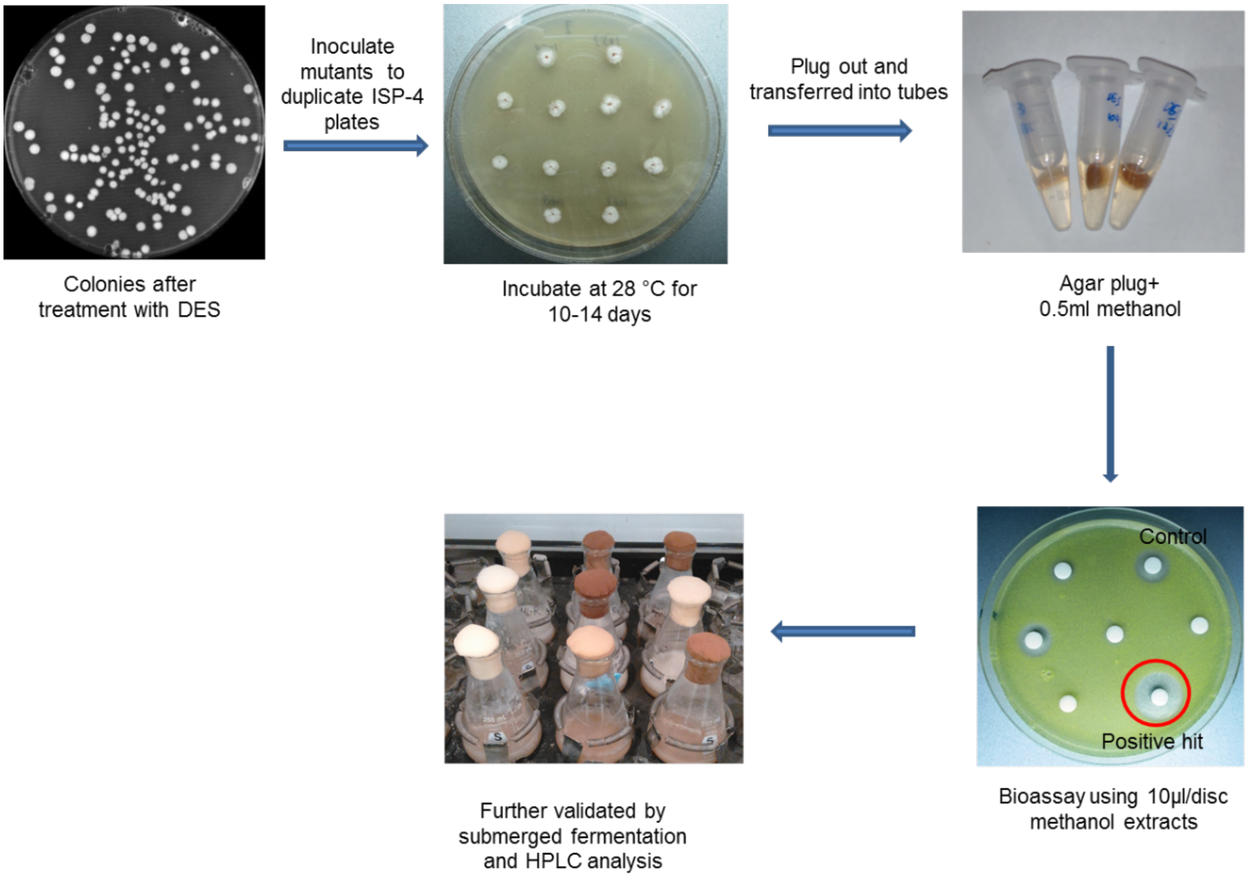

Figure S7. Inactivation of *ucmM* and *ucmP* by gene replacement in *S. uncialis* SB18002. (A) Generation of the Δ*ucmM* strain *S. uncialis* SB18008*.* Schematic diagram of the gene replacement of *ucmM* (via homologous recombination) with an apramycin resistance cassette containing origin of transfer (*oriT*). (B) Southern analysis of *S. uncialis* SB18008 (lane 1) and SB18002 (lane 2) using *Bam*HI digestion and DIG labelled-*ucmN* PCR fragment (342-bp probe). Lane 3 represents molecular weight marker. (C) Generation of the Δ*ucmP* strain *S. uncialis* SB18009*.* Schematic diagram of the gene disruption of *ucmP* by replacing it with the apramycin resistance cassette. (D) Southern analysis of *S. uncialis* SB18002 (lane 1) and SB18009 (lane 2) using *Bam*HI (Ba) digestion and DIG labelled-*ucmO* PCR fragment (228 bp probe).

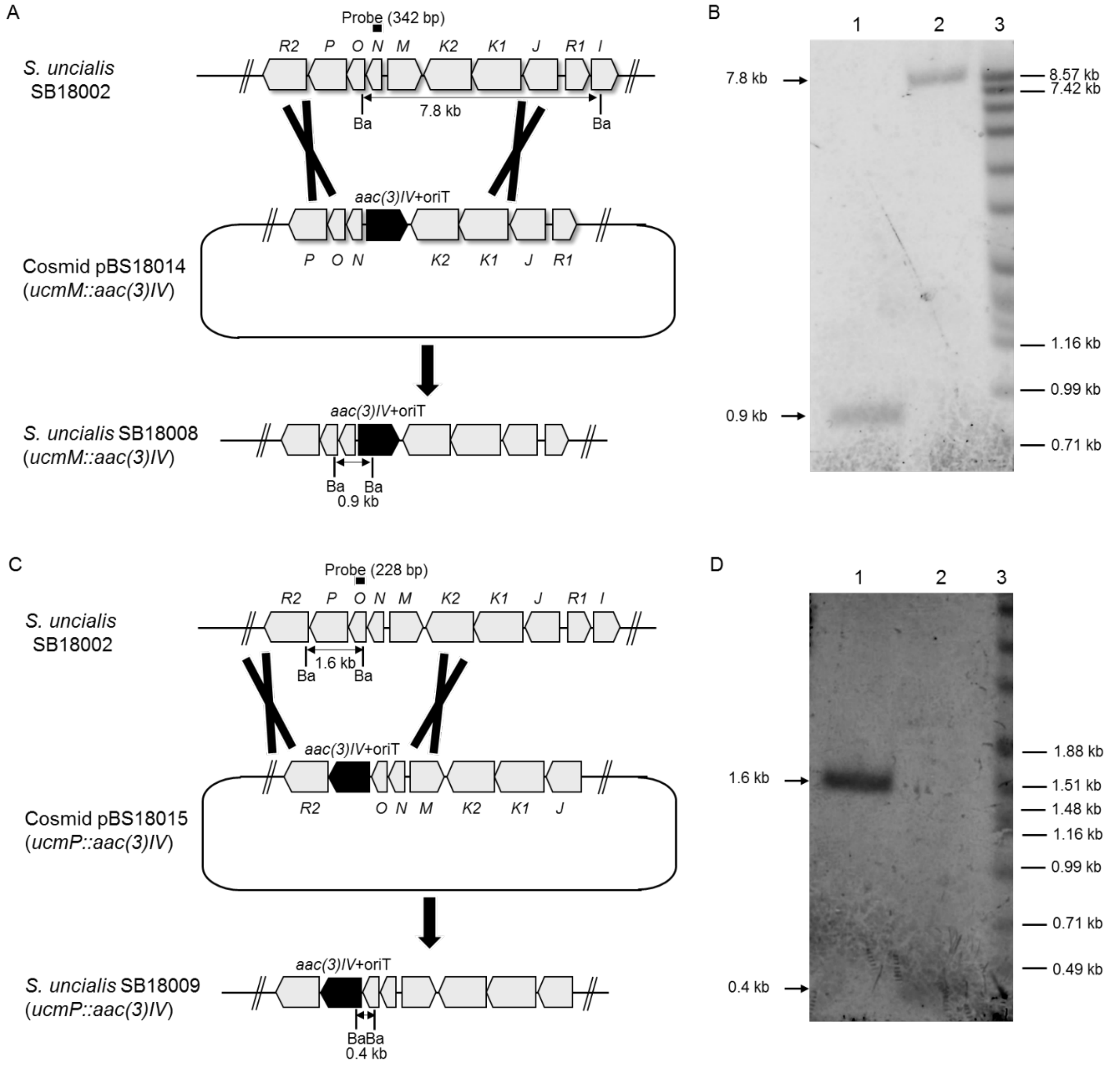

Figure S8. HR-ESI-MS spectrum of TNM B (calculated [M – H]⁻ ion for $C_{29}H_{18}NO_7^-$ at *m/z* 492.1088 and [M + COOH]⁻ ion for $C_{30}H_{20}NO_9^-$ at *m/z* 538.1144, respectively).

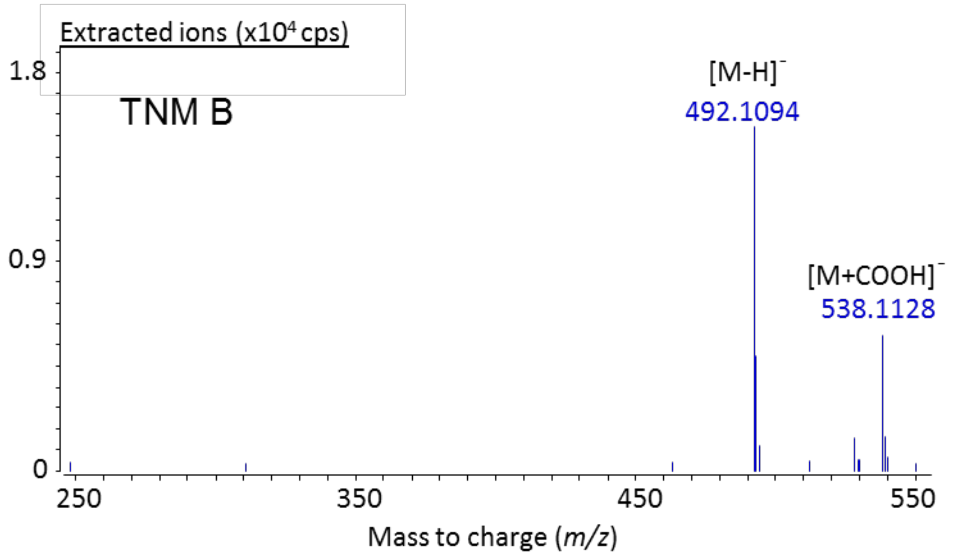

Figure S9. ^{1}H NMR (700 MHz) and ^{13}C NMR (175 MHz) spectra of TNM B in acetone-d_6

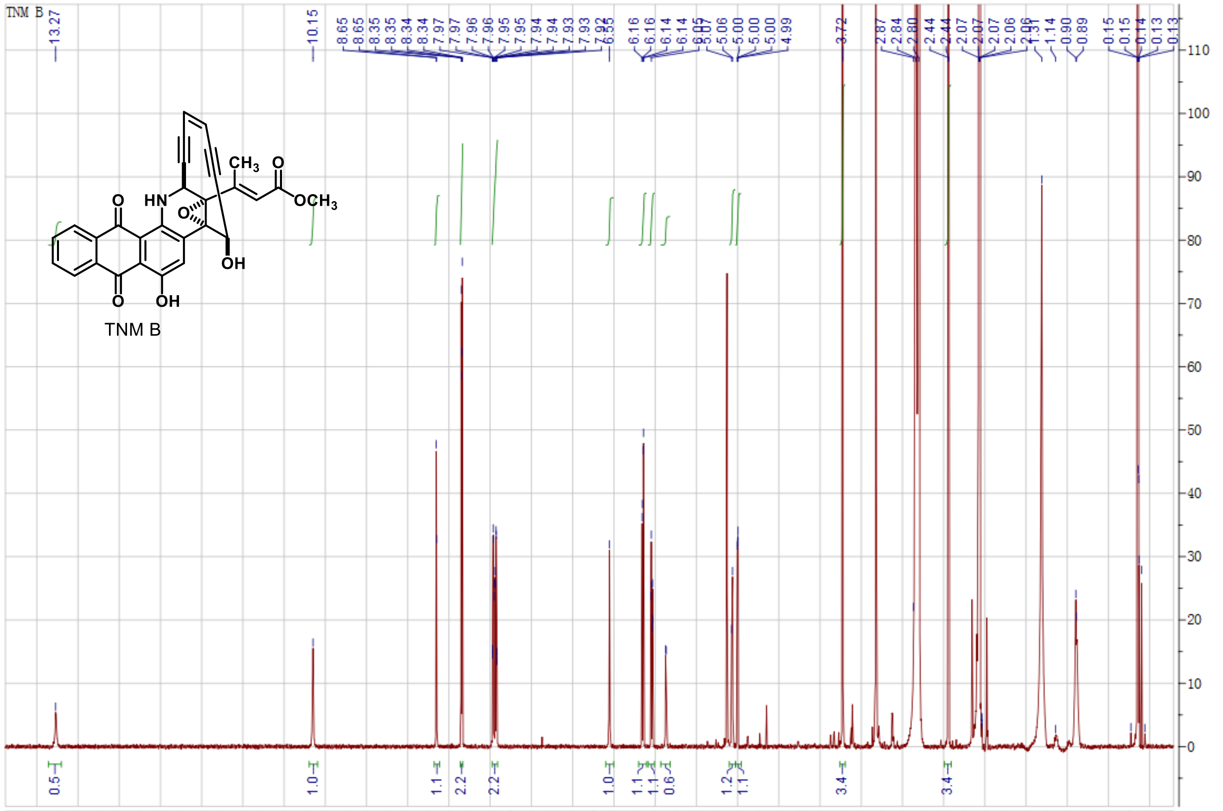

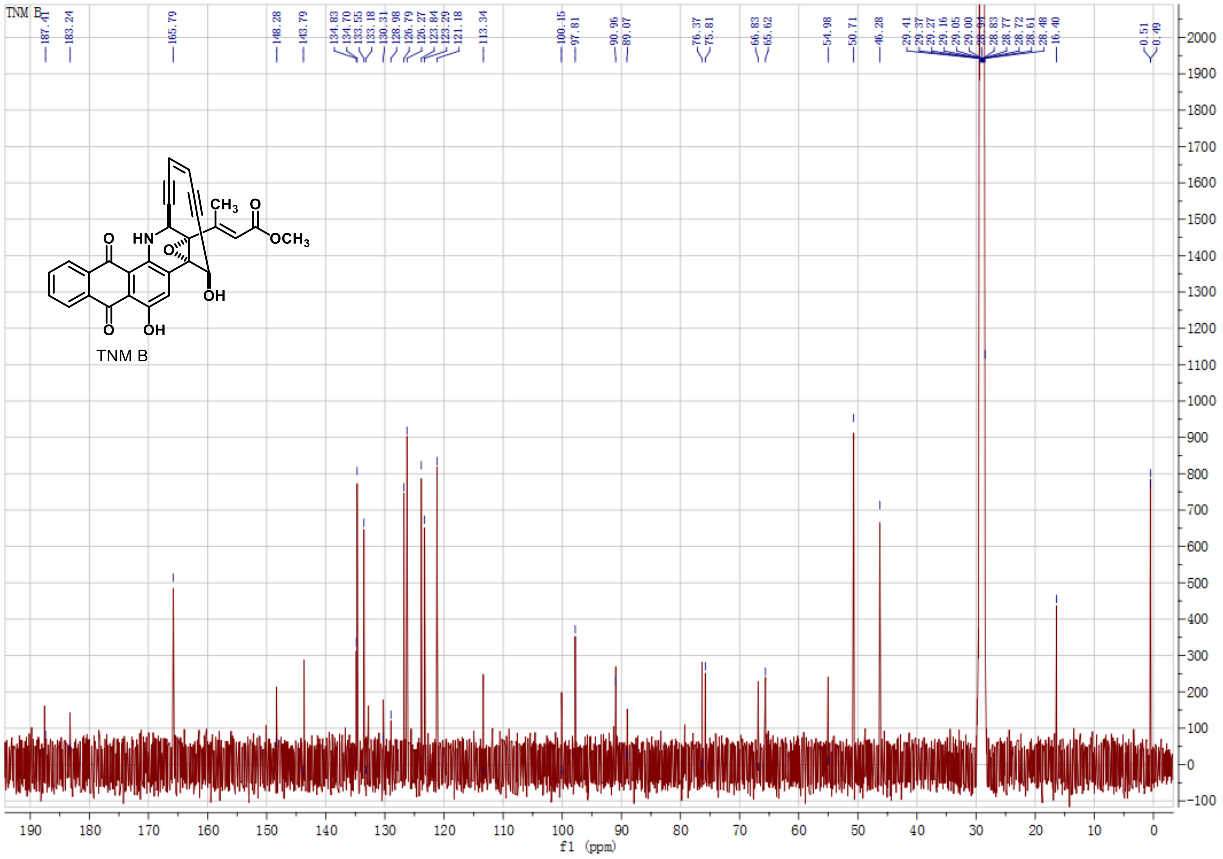

Supplementary references

Chen, Y., Yin, M., Horsman, G. P., Huang, S., & Shen, B. (2010) Manipulation of pathway regulation in Streptomyces globisporus for overproduction of the enediyne antitumor antibiotic C-1027. The Journal of Antibiotics, 63:482–485. https://doi.org/10.1038/ja.2010.55

Davies, J., Wang, H., Taylor, T., Warabi, K., Huang, X. H., & Andersen, R. J. (2005) Uncialamycin, a new enediyne antibiotic. Organic Letters, 7:5233−5236. https://doi.org/10.1021/ol052081f

Gregory, M. A., Till, R., & Smith, M. C. (2003) Integration site for *Streptomyces* phage φBT1 and development of site-specific integrating vectors. Journal of Bacteriology, 185:5320–5323. https://doi.org/10.1128/JB.185.17.5320-5323.2003

Gust, B., Challis, G. L., Fowler, K., Kieser, T., & Chater, K. F. (2003) PCR-targeted *Streptomyces* gene replacement identifies a protein domain needed for biosynthesis of the sesquiterpene soil odor geosmin. Proceedings of the National Academy of Sciences, 100: 1541−1546. https://doi.org/10.1073/pnas.0337542100

Hindra, Yang, D., Teng, Q., Dong, L. B., Crnovčić, I., Huang, T., Ge, H., & Shen, B. (2017) Genome mining of *Streptomyces mobaraensis* DSM40847 as a bleomycin producer providing a biotechnology platform to engineer designer bleomycin analogues. Organic letters, 19:1386–1389. https://doi.org/10.1021/acs.orglett.7b00283

Lam, K. S., Titus, J. A., Dabrah, T. T., Kimball, D. L., Veitch, J. M., Gustavson, D. R., Compton,, B. J., Matson, J. A., Forenza, S., Ross, J., & Miller, D. (1992) Improved processes for the production and isolation of dynemicin A and large-scale fermentation in a 10000-liter fermentor. Journal of Industrial Microbiology, 11:7–12. https://doi.org/10.1007/BF01583725

MacNeil, D. J., Gewain, K. M., Ruby, C. L., Dezeny, G., Gibbons, P. H., & MacNeil, T. (1992) Analysis of *Streptomyces avermitilis* genes required for avermectin biosynthesis utilizing a novel integration vector. Gene, 111: 61-68. https://doi.org/10.1016/0378-1119(92)90603-m

Shirling, E. T., & Gottlieb, D. (1966) Methods for characterization of *Streptomyces* species. International Journal of Systematic Bacteriology, 16:313–340. https://doi.org/10.1099/00207713-16-3-313

Yan, X., Chen, J. J., Adhikari, A., Teijaro, C. N., Ge, H., Crnovcic, I., Chang, C. Y., Annaval, T., Yang, D., Rader, C., & Shen, B. (2018) Comparative studies of the biosynthetic gene clusters for anthraquinone-fused enediynes shedding light into the tailoring steps of tiancimycin biosynthesis. Organic Letters, 20:5918–5921. https://doi.org/10.1021/acs.orglett.8b02584

Yan, X., Chen, J.-J., Adhikari, A., Yang, D., Crnovcic, I., Wang, N., Chang, C. -Y., Rader, C. & Shen, B. (2017) Genome mining of *Micromonospora yangpuensis* DSM 45577 as a producer of an anthraquinone-fused enediyne. Organic Letters, 19:6192−6195. https://doi.org/10.1021/acs.orglett.7b03120

Yan, X., Ge, H., Huang, T., Hindra, Yang, D., Teng, Q., Crnovčić, I., Li, X., Rudolf, J. D., Lohman, J. R., Gansemans, Y., Zhu, X., Huang, Y., Zhao, L.-X., Jiang, Y., Nieuwerburgh, F. V., Rader, C., Duan, Y., &

Shen, B. (2016) Strain prioritization and genome mining for enediyne natural products. mBio, 7:e02104−16. https://doi.org/10.1128/mBio.02104-16
